# Application of Serum CA125, HE4, ROMA, and CPH‐I in the Preoperative Differentiation of Ovarian Tumors: A Diagnostic Accuracy Study

**DOI:** 10.1002/jcla.70319

**Published:** 2026-08-11

**Authors:** Weiming Feng, Chunying Zhang, Lingxin Meng, Yifei Chen, Haiyun Yu, Yanhong Zhai, Zheng Cao

**Affiliations:** ^1^ Department of Laboratory Medicine, Beijing Obstetrics and Gynecology Hospital Capital Medical University, Beijing Maternal and Child Health Care Hospital Beijing China; ^2^ Center of Clinical Mass Spectrometry, Beijing Obstetrics and Gynecology Hospital Capital Medical University. Beijing Maternal and Child Health Care Hospital Beijing People's Republic of China; ^3^ Department of Pathology, Beijing Obstetrics and Gynecology Hospital Capital Medical University. Beijing Maternal and Child Health Care Hospital Beijing People's Republic of China

**Keywords:** biomarkers, CA125, Copenhagen index, diagnosis, HE4, ovarian tumor, ROMA

## Abstract

**Objective:**

This study aimed to compare the diagnostic accuracy of four serum biomarkers—carbohydrate antigen 125 (CA125), human epididymis protein 4 (HE4), the Risk of Ovarian Malignancy Algorithm (ROMA), and the Copenhagen Index (CPH‐I)—for the preoperative discrimination of benign/borderline from malignant ovarian cancer, with particular attention to menopausal status.

**Methods:**

A retrospective study enrolled 345 patients with histologically confirmed ovarian tumors (187 benign/borderline; 158 malignant). Serum CA125 and HE4 were measured and reported by the electrochemiluminescence immunoassays, along with the calculated indices of ROMA and CPH‐I. Diagnostic performance was assessed by the area under the curve (AUC), sensitivity, and specificity, respectively.

**Results:**

Among the 158 ovarian tumors patients, CPH‐I demonstrated superior discriminative capacity with the highest AUC (0.924), followed by ROMA (0.916), HE4 (0.897), and CA125 (0.865). Menopausal stratification identified CPH‐I as optimal for premenopausal women (AUC 0.886) and ROMA for postmenopausal women (AUC 0.928). ROMA exhibited the highest overall sensitivity (84.8%), whereas CA125 achieved the highest overall specificity (94.1%). In premenopausal patients, HE4 demonstrated exceptional specificity (98.6%), while postmenopausal women showed equivalent specificity between HE4 and CPH‐I (both 97.8%). In combined panels, CA125 + HE4 + CPH‐I yielded the highest overall accuracy (AUC 0.925), and CA125 + HE4 + ROMA provided optimal postmenopausal performance (AUC 0.930).

**Conclusions:**

CPH‐I demonstrated favorable diagnostic performance compared with conventional biomarkers. Our findings advocate for a menopausal status‐guided clinical algorithm, utilizing a CPH‐I‐based panel for premenopausal women and a ROMA‐based panel for postmenopausal women to maximize diagnostic precision.

## Graphical abstract

This study evaluated the diagnostic performance of CA125, HE4, ROMA, and CPH‐I in 345 patients with histologically confirmed ovarian tumors. CPH‐I showed favorable diagnostic performance overall and was particularly valuable in premenopausal women, whereas ROMA‐based strategies demonstrated optimal performance in postmenopausal women. A menopausal status‐guided biomarker selection strategy may improve preoperative differentiation of ovarian tumors and requires further prospective validation.

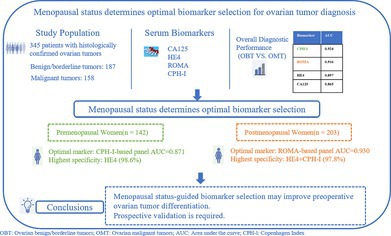

**Abbreviations**
AUC: area under the curve
CA125: Carbohydrate Antigen 125
CPH‐I: Copenhagen index
EOC: epithelial ovarian cancer
FIGO: International Federation of Gynecology and Obstetrics
HE4: human epididymal protein 4
MCO: metastatic cancer in the ovary
Non‐EOC: non‐epithelial ovarian cancer
NPV: negative predictive value
OBT: ovarian benign tumors
OC: ovarian cancer
OMT: ovarian malignant tumors
PPV: positive predictive value
ROC: receiver operating characteristic curve
ROMA: risk of ovarian malignancy algorithm
SEN: sensitivity
SPE: specificity
WHO: World Health Organization

## 1 Introduction

Ovarian cancer (OC) ranks as the third most common but most lethal gynecologic malignancy, owing largely to its non‐specific and atypical early symptoms and late diagnosis, resulting in over 75% of OC patients being diagnosed at advanced stages (IIIIV) with poor long‐term survival [1, 2]. Early‐stage diagnosis and treatment significantly impact the prognosis and survival rate of patients, underscoring an urgent need for reliable diagnostic biomarkers.

Serum tumor biomarkers are pivotal in the preoperative evaluation of OC. Carbohydrate Antigen 125 (CA125), the first clinically validated OC biomarker, exhibits limited sensitivity and low specificity as elevated levels are frequently observed in benign conditions such as endometriosis, pelvic inflammatory disease, and other non‐gynecological malignancies [3, 4, 5]. Human Epididymal Protein 4 (HE4) has emerged in recent years as a valuable alternative, exhibiting enhanced sensitivity and specificity, particularly in early‐stage disease and premenopausal patients, with minimal expression in benign ovarian pathologies [6, 7]. However, HE4 levels may also increase in other pathological conditions, such as lung cancer, endometrial cancer, or renal failure [5, 8], and their concentration varies with age, complicating the establishment of universal reference thresholds. To improve diagnostic accuracy, multivariate models such as the Risk of Ovarian Malignancy Algorithm (ROMA), which combines HE4, CA125, and menopausal status, are used to assess the risk of OC and has been approved by the FDA [9, 10], While the CPH‐I model (incorporating CA125, HE4, and age) enhances diagnostic accuracy through integration of this objectively quantifiable covariate [11, 12].

Accurate differentiation of malignant from benign or borderline OC is essential since management varies dramatically, from conservative to aggressive intervention. Currently, there is limited research on the use of CA125, HE4, ROMA, and CPH‐I to differentiate OC from non‐OC, particularly in women with varying menopausal statuses. This study aims to assess and compare the diagnostic performance of these biomarkers individually and in combination for discriminating between benign/borderline and malignant ovarian tumors, with particular emphasis on their utility in premenopausal and postmenopausal subgroups. Our findings seek to contribute to optimized early‐stage diagnosis, enable timely therapeutic intervention, and improve prognostic outcomes.

## 2 Materials and Methods

### 2.1 Study Population and Design

A retrospective study included 345 women diagnosed with histologically confirmed ovarian tumors at our hospital between January 1, 2024, and December 31, 2024. Based on final histopathological diagnosis, these were categorized as: benign tumors (*n* = 177), borderline tumors (*n* = 10), and malignant tumors (*n* = 158). Borderline tumors were grouped with benign lesions because they are generally considered tumors with low malignant potential. Metastatic ovarian cancer referred to ovarian metastases originating from extra‐ovarian primary tumors. Utilizing the International Federation of Gynecology and Obstetrics (FIGO) staging criteria (2014), they were further classified into an early‐stage ovarian malignant tumor group (Stage I and Stage II, *n* = 48) and a late‐stage ovarian malignant tumor group (Stage III and Stage IV, *n* = 110). According to the World Health Organization (WHO) ovarian tumor histological classification (2020), they were divided into an epithelial ovarian cancer group (EOC, 132 cases) and a non‐epithelial ovarian cancer group (Non‐EOC, 187 cases).

### 2.2 Specimen Collection and Detection

Clinicopathological data, including CA125, HE4, age, menopausal status, histopathology, and staging, were abstracted from the electronic medical record system, with preoperative serological biomarker quantification performed universally. Serum CA125 and HE4 levels were measured using the Roche Elecsys CA125 II and Elecsys HE4 diagnostic kits (made in Germany) on the Roche Cobas E601 analyzer via electrochemiluminescence. The detection ranges for CA125 and HE4 were 0–5000 U/mL and 15–1500 pmol/L, respectively. The index values for ROMA and CPH‐I for all patients were calculated using the algorithms published by Moore, McMeekin et al. [9]. All tests were conducted with the manufacturer's instructions. For sample testing, daily internal quality control was performed at both normal and pathological concentrations using quality control products. This study was approved by the Research Ethics Committee (2024‐KY‐083‐01).

Based on the pathological results, two research groups were defined. The OBT group consisted of women with benign and borderline epithelial ovarian tumors. The OMT group included women diagnosed with EOC or malignant Brenner tumor (MCO). This grouping was based on differences in clinical triage for later stages and surgical arrangements, with patients diagnosed with EOC or MCO being given priority.

### 2.3 Statistical Analysis

Statistical analyses were performed using SPSS software (version 27.0; IBM Corp., Armonk, NY, USA). Continuous variables were expressed as mean ± standard deviation or median (interquartile range), and categorical variables as frequencies and percentages. Group comparisons were performed using the independent‐sample *t*‐test or one‐way ANOVA for normally distributed data, and the Mann–Whitney *U* or Kruskal–Wall test for non‐normally distributed variables. Count data were presented as numbers and percentages. A *p*‐value < 0.05 was considered to indicate a statistically significant difference between data groups. The sensitivity (SEN), specificity (SPE), positive predictive value (PPV), and negative predictive value (NPV) of CA125, HE4, ROMA, and CPH‐I in the diagnosis of ovarian tumors were calculated both individually and in combination. The receiver operating characteristic curve (ROC) was plotted, and the area under the curve (AUC), along with the 95% confidence interval, was computed.

## 3 Results

### 3.1 General Clinical Characteristics of Patients

A total of 345 patients were included in this study (Table 1). The OBT group comprised 187 patients (age range: 11–75 years; mean ± SD: 42.69 ± 14.84 years), while the OMT group consisted of 158 patients (age range: 15–86 years; mean ± SD: 56.04 ± 10.50 years). A significant age difference was observed between groups (*p* < 0.05), with the OMT group being older than the OBT group. Regarding menopausal status, the OBT group included 141 premenopausal and 46 postmenopausal patients, whereas the OMT group comprised 44 premenopausal and 114 postmenopausal patients. The distribution of menopausal status differed significantly between the two groups (*p* < 0.05), with a higher proportion of postmenopausal patients in the OMT group. Additionally, pathological staging of the OMT group revealed 48 cases at the FIGO stage I and II, 110 cases at stage III and IV. Advanced‐stage disease (III and IV) predominated in the OMT group (69.6%), with a significant difference in stage distribution compared to OBT (*p* < 0.001). As shown in Table 2, pathological classification revealed: Group A: benign or borderline epithelial tumors (*n* = 187, 54.20%); Group B: malignant tumors (*n* = 158, 45.80%) comprising 132 primary epithelial OC and 26 metastatic ovarian cancer. Among primary OC, serous carcinoma was most frequent (59.1%).

**TABLE 1:** Clinical information.

	Total (*n* = 345)	OBT (*n* = 187)	OMT (*n* = 158)
**Age**	48.81 ± 14.62	42.69 ± 14.84	56.04 ± 10.50
**Menstrual status**			
Premenopausal	185 (53.6%)	141 (75.4%)	44 (27.8%)
Postmenopausal	160 (46.4%)	46 (24.6%)	114 (72.2%)
**FIGO stage**			
I	NA	NA	35 (22.15%)
II	NA	NA	13 (8.23%)
III	NA	NA	89 (56.33%)
IV	NA	NA	21 (13.29%)

Abbreviations: OBT, Benign/borderline ovarian tumor; OMT, Malignant ovarian tumor.

**TABLE 2:** Histopathology.

Histopathology	*N* = 345
**Benign**	177 (51.3%)
Benign epithelial ovarian tumor^a^	99 (55.9%)
Endometriosis	33 (18.6%)
Leiomyoma	10 (5.6%)
Mature teratoma	21 (11.9%)
Fibroma	7 (4.0%)
Other sex cord stromal tumors	4 (2.3%)
Inflammatory lesions	3 (1.7%)
**Epithelial borderline tumors**	10 (2.9%)
**Epithelial ovarian cancer (EOC)**	132 (38.3%)
Serous carcinoma	78 (59.1%)
Mucous carcinoma	14 (10.6%)
Clear‐cell carcinoma	17 (12.9%)
Endometrioid carcinoma	10 (7.6%)
Undifferentiated carcinoma	13 (9.8%)
**Metastatic cancer to the ovary** ^b^	26 (7.5%)

*Note:* ^a^Benign epithelial ovarian tumors included serous cystadenoma, mucinous cystadenoma, etc. ^b^Metastatic tumors originated from colorectal, gastric, breast and other primary tumors.

### 3.2 Comparison of Serum Biomarker Levels

As indicated in Table 3, serum levels of CA125, HE4, ROMA, and CPH‐I were significantly higher in the OMT group compared with the OBT group (Mann–Whitney *U* test; all *p* < 0.05). When stratified by menopausal status (Table 4), in the OBT group, all four biomarkers (CA125, HE4, ROMA, CPH‐I) exhibited significant differences between premenopausal and postmenopausal patients (*p* = 0.001, *p* = 0.001, *p* = 0.001, *p* = 0.036, respectively), suggesting that menopausal status strongly influences biomarker levels in benign ovarian tumors. In contrast, in the OMT group, significant menopausal differences related to menopausal status were observed only for ROMA (*p* = 0.012) and CPH‐I (*p* = 0.030), whereas CA125 (*p* = 0.447) and HE4 (*p* = 0.056) showed no significant variation by menopausal status. Moreover, as indicated in Table 5, serum levels of CA125, HE4, ROMA, and CPH‐I were all significantly elevated in patients with advanced‐stage OMT (FIGO stage III and IV) compared with those at early‐stage patients (FIGO stage I and II) (Mann–Whitney U test; all *p*‐values < 0.05).

**TABLE 3:** Comparison of CA125, HE4, ROMA, and CPH‐I levels in the OBT and OMT groups.

	OBT (*n* = 187)	OMT (*n* = 158)	*p*
CA125 (U/ml)	17.77 (10.69, 40.56)	192.65 (54.94, 697.08)	< 0.05
HE4 (pmol/L)	48.24 (42.46, 53.74)	191.10 (84.91, 431.70)	< 0.05
ROMA (%)	7.03 (5.21, 9.66)	75.68 (35.61, 94.32)	< 0.05
CPH‐I (%)	1.31 (0.67, 2.39)	48.24 (9.00, 90.98)	< 0.05

Abbreviations: OBT, Benign/borderline ovarian tumor; OMT, Malignant ovarian tumor.

**TABLE 4:** Comparison of CA125, HE4, ROMA, and CPH‐I levels before and after menopause in the OBT and OMT groups.

	OBT	*p*	OMT	*p*
CA125 (U/ml)		0.001		0.447
Premenopausal	24.78 (13.30, 46.77)		140.05 (49.22, 481.33)	
Postmenopausal	9.54 (6.44, 19.72)		214.65 (60.52, 737.18)	
HE4 (pmol/L)		0.001		0.056
Premenopausal	45.89 (40.35, 51.59)		163.90 (50.65, 353.73)	
Postmenopausal	53.22 (47.81, 66.03)		196.85 (111.68, 447.28)	
ROMA (%)		0.001		0.012
Premenopausal	6.34 (4.90, 8.52)		61.64 (7.92, 91.01)	
Postmenopausal	9.66 (6.40, 17.27)		80.98 (44.13, 94.68)	
CPH‐I (%)		0.036		0.030
Premenopausal	1.29 (0.60, 2.31)		31.04 (3.58, 87.29)	
Postmenopausal	1.54 (0.85, 3.38)		60.12 (16.18, 93.65)	

Abbreviations: OBT, Benign/borderline ovarian tumor; OMT, Malignant ovarian tumor.

**TABLE 5:** Comparison of CA125, HE4, ROMA, and CPH‐I levels in early and advanced ovarian malignancies.

	Early‐stage ovarian malignant tumor group (I and II)	Advanced‐stage ovarian malignant tumor group (III and IV)	*p*
CA125 (U/ml)	58.31 (18.91, 175.43)	303.85 (114.68, 919.55)	< 0.001
HE4 (pmol/L)	81.49 (47.15, 210.48)	249.50 (128.48, 587.08)	< 0.001
ROMA (%)	24.19 (8.25, 72.88)	84.95 (59.01, 96.45)	< 0.001
CPH‐I (%)	6.40 (1.98, 39.70)	70.50 (31.87, 96.15)	< 0.001

### 3.3 Diagnostic Efficacy for Differentiating Benign and Malignant Tumors

**FIGURE 1:**
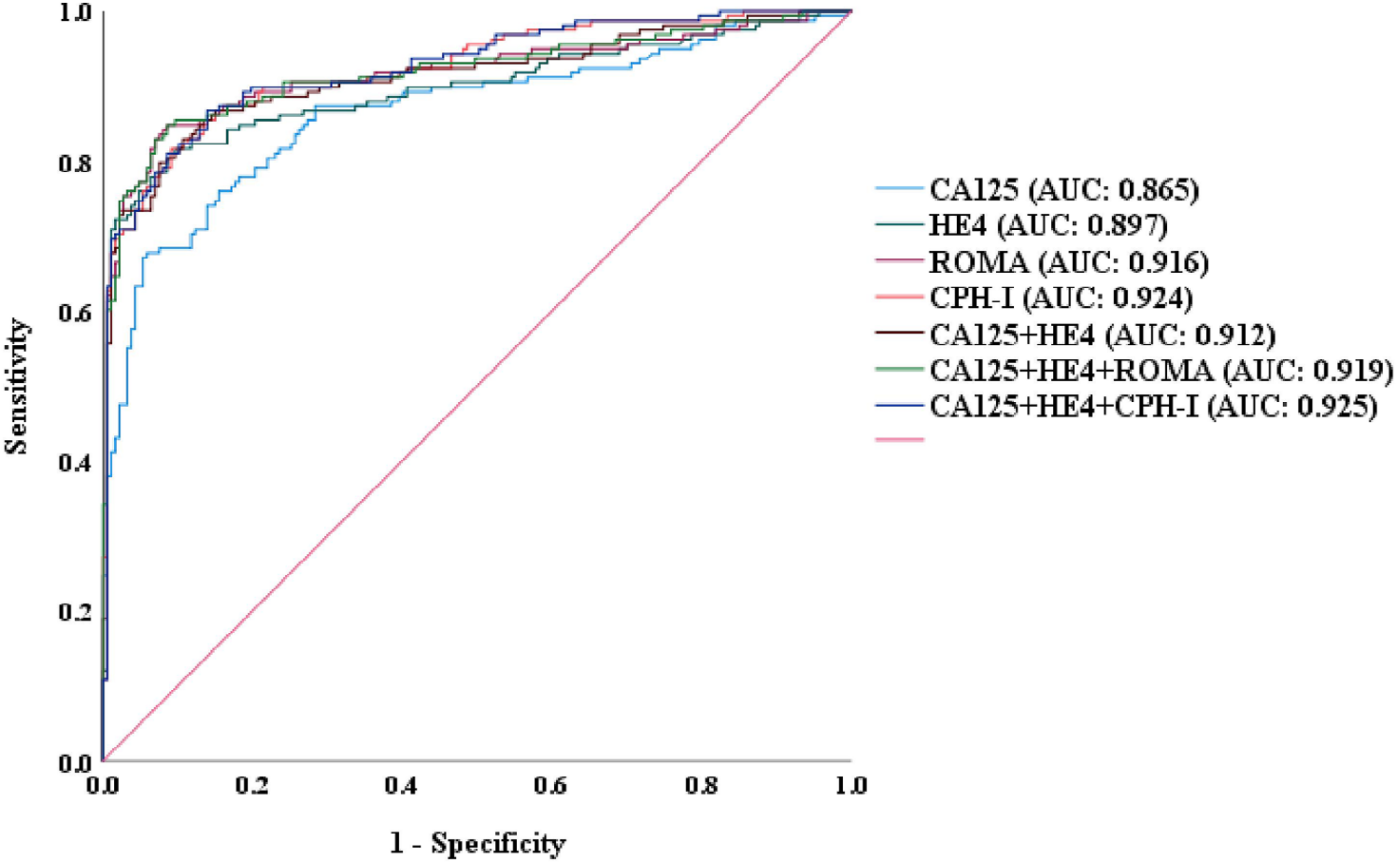
ROC curves of CA 125, HE4, ROMA, CPH‐I, and the combination test for distinguishing between benign and malignant ovarian tumors.

ROC analysis demonstrated significant diagnostic performance for all individual biomarkers in distinguishing OBT from OMT (all AUCs > 0.85, all *p* < 0.05; Figure 1, Table S1). Among single markers, CPH‐I showed the highest overall discriminatory ability (AUC = 0.924), followed by ROMA (AUC = 0.916), HE4 (AUC = 0.897), and CA125 (AUC = 0.865). ROMA exhibited the highest sensitivity (84.80%), while CA125 showed the highest specificity (94.10%). Positive predictive value was greatest for CA125 and decreased in the following order: ROMA > CPH‐I > HE4, whereas negative predictive value was highest for ROMA and decreased in the following order: CPH‐I > HE4 > CA125.

**FIGURE 2:**
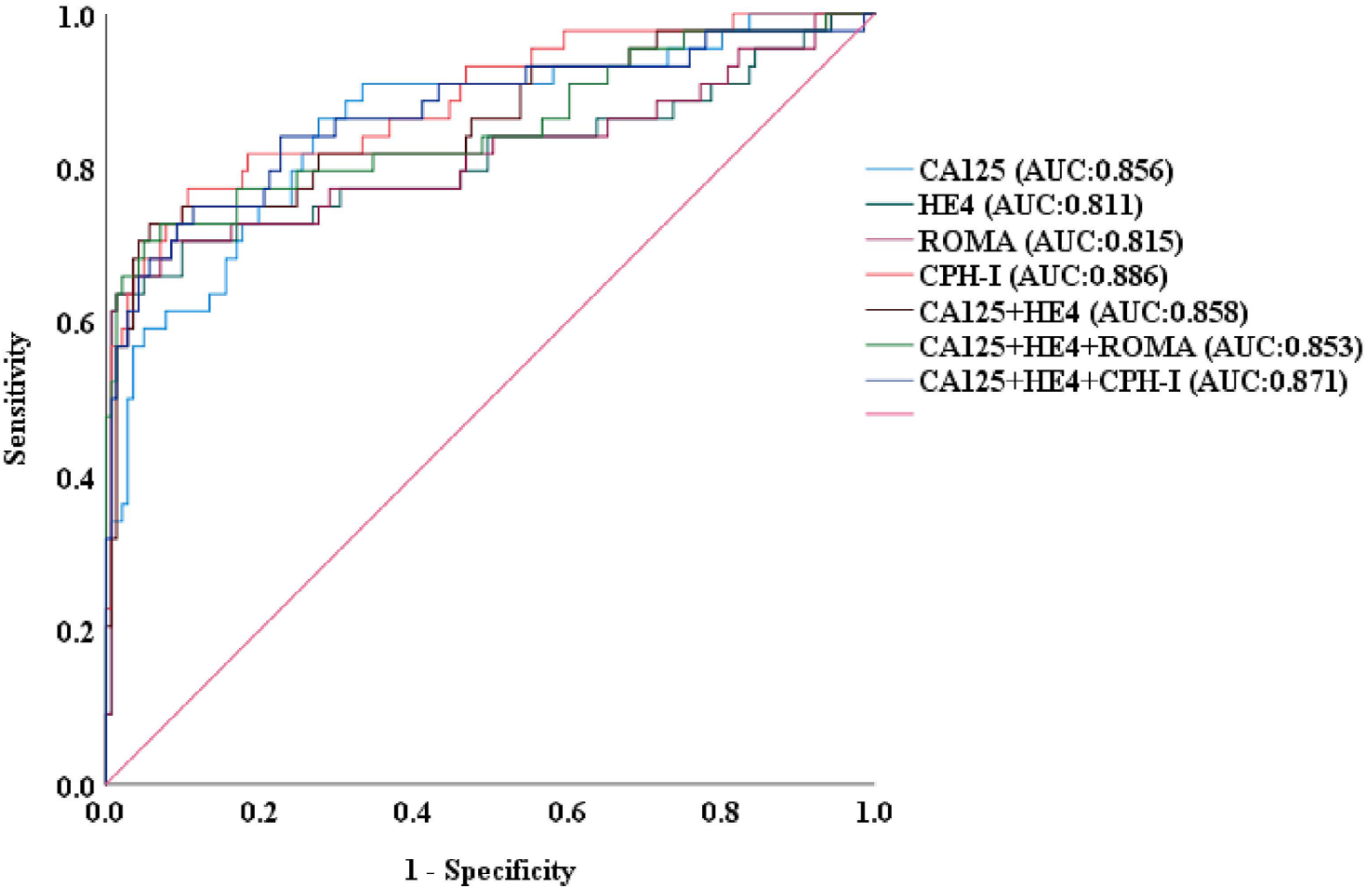
ROC curves of premenopausal CA125, HE4, ROMA, CPH‐I, and combined tests for distinguishing benign and malignant ovarian tumors.

**FIGURE 3:**
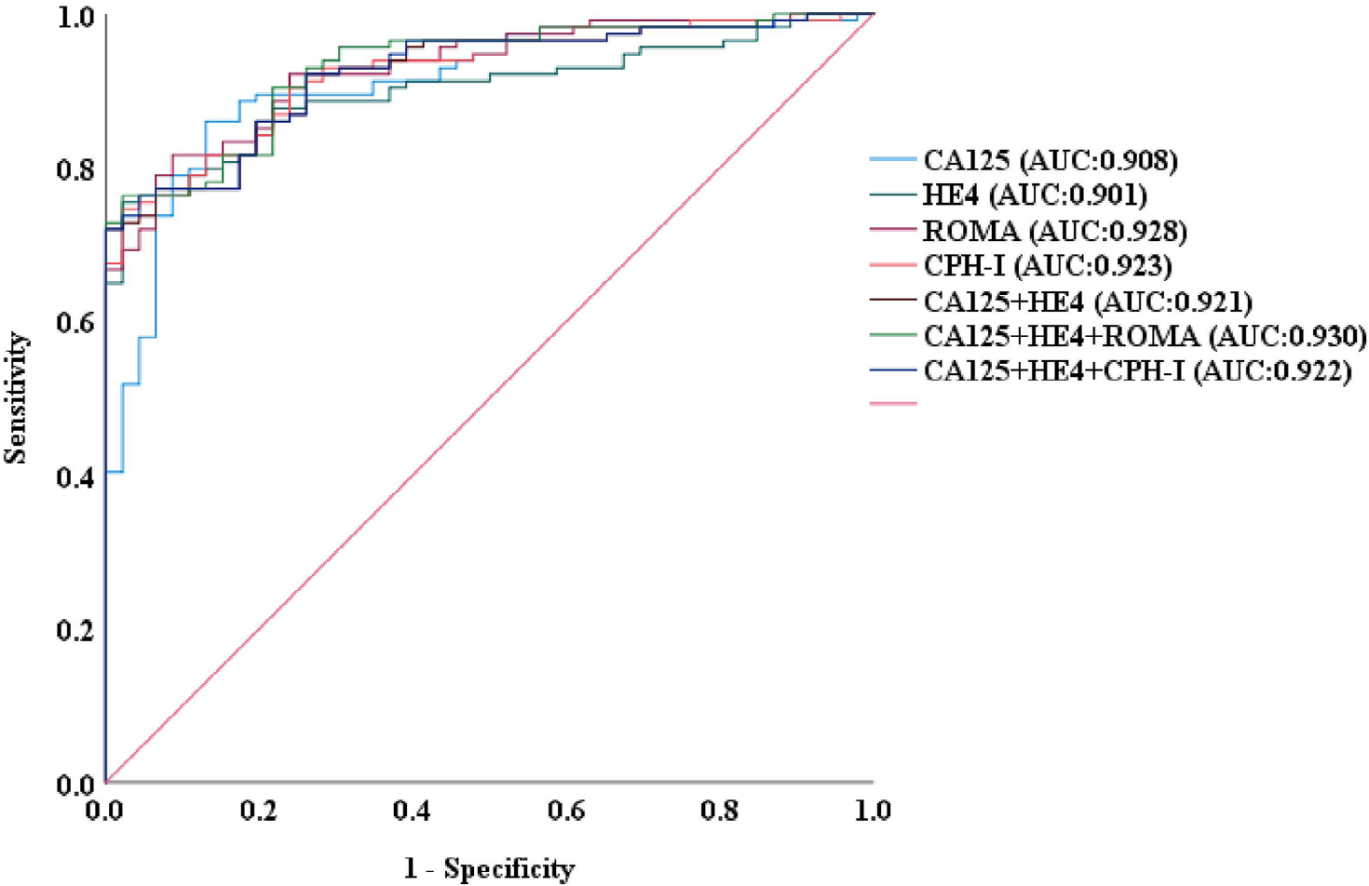
ROC curves of postmenopausal CA125, HE4, ROMA, CPH‐I, and combination detection for differentiating between benign and malignant ovarian tumors.

ROC curve analyses stratified by menopausal status are shown in Figures 2 and 3. In premenopausal women, CPH‐I achieved the highest AUC (0.886), whereas ROMA demonstrated the best diagnostic performance in postmenopausal women (AUC = 0.928). CA125 showed the highest sensitivity in both premenopausal (84.60%) and postmenopausal (86.00%) subgroups. Specificity was highest for HE4 in premenopausal women (98.6%), while in postmenopausal women, both HE4 and CPH‐I reached 97.8%, surpassing those of CA125 (87.00%) and ROMA (91.30%).

Optimal cut‐off values derived from the Youden index were: CA125 at 87.93 U/mL (42.17 U/mL premenopausal; 33.72 U/mL postmenopausal), HE4 at 67.28 pmol/L (82.09 pmol/L premenopausal; 111.90 pmol/L postmenopausal), ROMA at 16.42% (16.26% premenopausal; 35.40% postmenopausal), and CPH‐I at 4.51% (3.47% premenopausal; 18.28% postmenopausal). Of note, these thresholds were derived within our single‐center cohort and should be considered exploratory; external validation is required before clinical application.

In combined‐indicator diagnosis, CA125 + HE4 + CPH‐I achieved the highest AUC (0.925) and sensitivity (86.70%), whereas CA125 + HE4 + ROMA showed the highest specificity (91.4%). In subgroup analysis, CA125 + HE4 + CPH‐I performed best in premenopausal women (AUC = 0.871), while CA125 + HE4 + ROMA performed best in postmenopausal women (AUC = 0.930). All combinations significantly outperformed individual markers (all *p* < 0.05).

### 3.4 Diagnostic Efficacy for Tumor Staging

**FIGURE 4:**
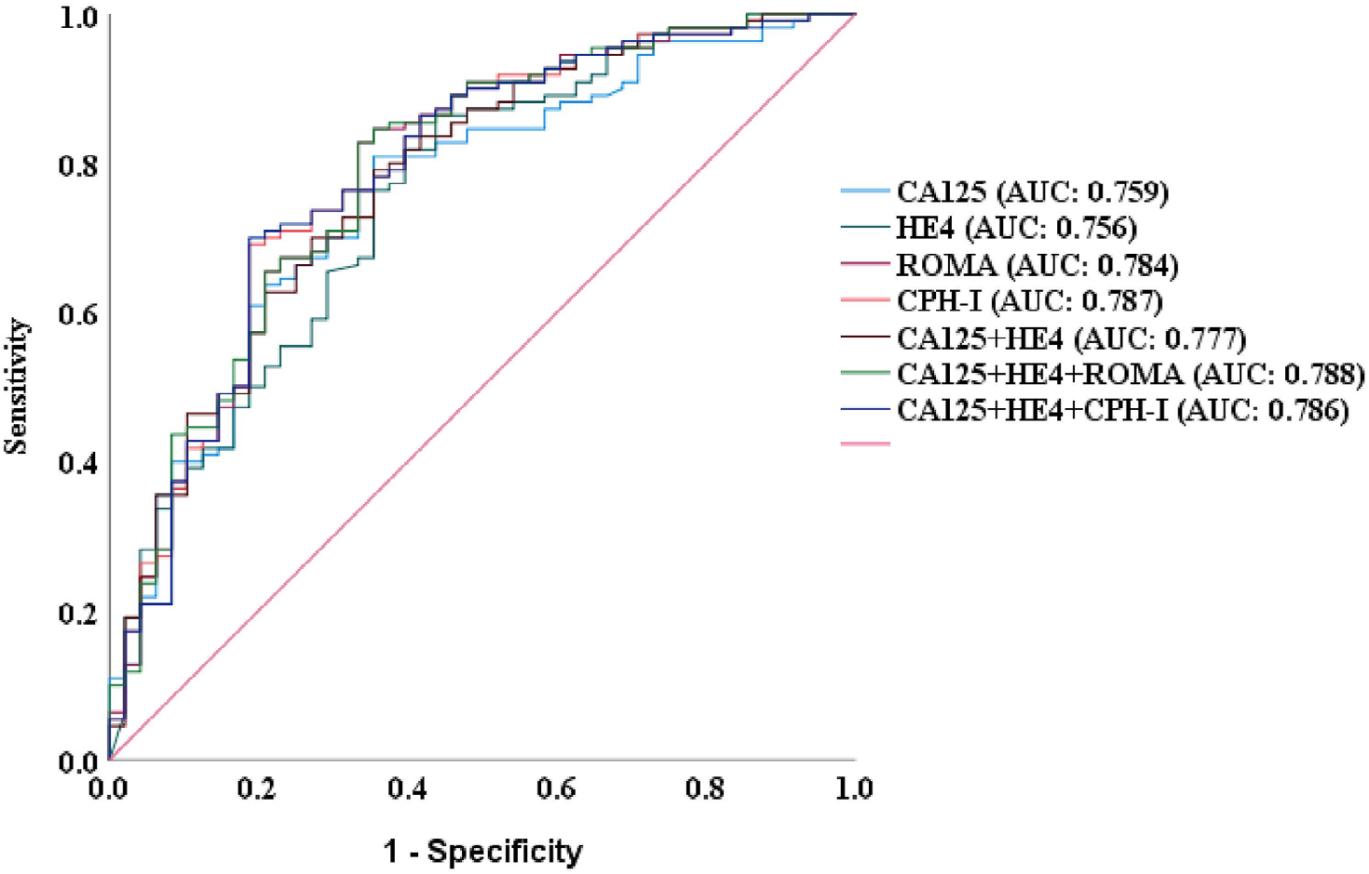
Comparative ROC Analysis of CA 125, HE4, ROMA, and CPH‐I with Multi‐marker algorithms for discriminating early‐stage from advanced ovarian tumors.

As shown in Figure 4 and Table S2, all biomarkers demonstrated significant discriminatory capability between early‐ and late‐stage OMT (all AUCs > 0.75, all *p* < 0.05). Among them, the CPH‐I demonstrated the highest AUC (0.787), closely followed by ROMA (0.784), CA125 (0.759), and HE4 (0.756). When the Youden index was maximized, the cut‐off values for differentiating early and late‐stage malignant tumors were determined as follows: CA125 (91.59 U/mL), HE4 (94.38 pmol/L), ROMA (46.33%), and CPH‐I (45.20%). HE4 showed the highest sensitivity (86.40%) but the lowest specificity (56.20%), whereas CPH‐I exhibited the opposite pattern with the highest specificity (81.20%) but the lowest sensitivity (69.10%). When biomarkers were combined, diagnostic performance improved: CA125 + HE4 + ROMA panel achieved the highest sensitivity (82.7%, AUC = 0.788), while CA125 + HE4 + CPH‐I reached the highest specificity (81.2%, AUC = 0.786). All combinations significantly outperformed individual biomarkers (all *p* < 0.05).

## 4 Discussion

Malignant ovarian tumors remain among the most lethal gynecologic malignancies, primarily due to the frequent late‐stage diagnosis associated with nonspecific symptoms and the absence of effective screening tools [13]. Due to the unclear pathogenesis and the difficulties in early diagnosis, over 70% of patients are already in the advanced stage at the time of diagnosis [14]. This underscores the urgent need for early detection tools and accurate risk stratification. This study comprehensively evaluated and compared the diagnostic performance of CA125, HE4, ROMA, and CPH‐I in distinguishing benign from malignant ovarian tumors, with a specific focus on menopausal status and tumor stage. Our key finding is that CPH‐I emerges as a superior individual biomarker, demonstrating a significantly higher specificity in premenopausal women, a population in which conventional biomarker interpretation is most challenging. This performance heterogeneity across menopausal strata reinforces the need for biomarker interpretation in the context of hormonal status.

Consistent with established epidemiological data, our cohort confirmed that age and menopausal status are strong predictors of malignancy, with 72.2% of malignant cases occurring in postmenopausal women. The average age of the malignant tumor group was significantly higher than the benign group, consistent with previous reports. Moreover, the proportion of advanced (III‐IV stage) patients in the OMT group (69.6%) highlights the clinical value of reliable biomarkers for early detection. Postmenopausal women warrant high suspicion for malignancy. The high proportion of advanced‐stage (FIGO III‐IV) diseases at diagnosis further underscores the critical need for improved diagnostic strategies.

All four biomarkers were significantly elevated in malignant compared with benign tumors, confirming their role in OC pathogenesis. Importantly, the expression levels of these markers correlated with tumor stage, with significantly higher levels in advanced‐stage malignancies, in agreement with Sandri et al. [15]. These results support their potential utility not only in diagnosis but also as indicators of tumor burden and aggressiveness. Importantly, menopausal status influenced biomarker performance. In benign tumors, all four markers differed significantly between pre‐ and postmenopausal women; however, in malignant tumors, only ROMA and CPH‐I were significantly affected, suggesting that CA125 and HE4 are less susceptible to hormonal influences once malignancy develops. In the benign group, all four markers differed significantly between pre‐ and postmenopausal women. However, in the malignant group, only ROMA and CPH‐I levels were significantly affected by menopausal status, suggesting that CA125 and HE4 may be less susceptible to hormonal influences in the malignant context. Consistent with previous findings [8, 16], the interpretation of serum HE4 and ROMA levels requires consideration of patient menopausal status. This observation reinforces the clinical value of CPH‐I and ROMA as more stable indicators across different physiological states.

ROC analysis further demonstrated the diagnostic advantage of CPH‐I (AUC 0.924), outperforming ROMA (0.916), HE4 (0.897), and CA125 (0.865). Subgroup analysis revealed that CPH‐I was most accurate in premenopausal women (AUC 0.886), whereas ROMA had superior performance in postmenopausal women (AUC 0.928). Although the sensitivity of CA125 was the highest before and after menopause (84.60% before menopause and 86.00% after menopause), its specificity was low, confirming limitations noted in previous studies [17].

Interestingly, the optimal cut‐off values derived from our cohort varied from the manufacturer's recommendations. For example, the optimal CA125 thresholds were lower in postmenopausal women (33.72 U/mL) and higher in premenopausal women (42.17 U/mL). HE4 thresholds were also lower than the recommended values, possibly due to inclusion of non‐epithelial ovarian malignancies in our cohort. The optimal ROMA cut‐offs (16.26% and 35.40%) were higher than previously reported, potentially due to sample size differences. Notably, CPH‐I demonstrated the lowest optimal threshold (4.51%), consistent with its high sensitivity at low expression levels. When evaluating staging discrimination, CPH‐I again achieved the highest AUC (0.787), with greater specificity (81.2%) compared with other markers, albeit at a modest sensitivity (69.1%). Taken together, CPH‐I exhibits the highest overall diagnostic value in predicting ovarian malignancies and their staging, with a lower rate of misdiagnosis and significant advantages. This is consistent with the report by Hogdall E [18]. These findings should be considered hypothesis‐generating and warrant confirmation in independent, prospective multi‐center cohorts before clinical implementation. Combined biomarker testing yielded incremental improvements, particularly in sensitivity and negative predictive value. The combination of CA125 + HE4 + CPH‐I yielded the highest overall AUC (0.925), particularly among premenopausal women (AUC 0.871), while CA125 + HE4 + ROMA performed best in postmenopausal women (AUC 0.930). However, compared with single markers, combined tests did not substantially enhance specificity or positive predictive value, suggesting that while panels may optimize sensitivity, their ability to reduce false positives remains limited. Similarly, the combinations of CA125 + HE4 + ROMA and CA125 + HE4 + CPH‐I only slightly improved staging discrimination, indicating that progression‐related biomarker elevation may reflect common biological mechanisms.

This study has several limitations. First, this was a retrospective single‐center study without external validation. Second, the optimal cut‐off values were derived using the Youden index and may be subject to overfitting. Third, these thresholds may not be directly generalizable to other populations. Finally, the inclusion of non‐epithelial and metastatic ovarian malignancies may have influenced biomarker performance.

## 5 Conclusion

In this retrospective cohort, CPH‐I demonstrated favorable overall diagnostic utility, especially in premenopausal women, while ROMA provided strong performance in postmenopausal women. The performance of biomarker combinations varied by menopausal status, with CA125 + HE4 + CPH‐I being optimal for premenopausal patients and CA125 + HE4 + ROMA for postmenopausal patients. These findings support the potential clinical utility of these biomarkers but should be considered exploratory. Our proposed cut‐off values require external validation in prospective multi‐center studies before clinical implementation.

## Author Contributions

All authors have accepted responsibility for the entire content of this manuscript. W.F., Y.Z., and Z.C. designed the research; W.F., C.Z., L.M., and Y.C. performed the experiments. W.F. and C.Z. analyzed the data; W.F., C.Z., L.M., Y.C., Y.Z., and Z.C. wrote and revised the manuscript. H.Y. contributed pathological consultation and histopathological review of ovarian tumors. All authors read and approved the final manuscript.

## Funding

This study was supported by the National Key Research and Development Program of China (2023YFC3402902) and Beijing High Innovation Plan‐Leading Talent Projects (G202511060).

## Ethics Statement

Research involving human subjects complied with all relevant national regulations and institutional policies following the tenets of the Helsinki Declaration (as revised in 2013). Ethical approval (2024‐KY‐083‐01) was obtained from the Ethics Committee of Beijing Obstetrics and Gynecology Hospital, Capital Medical University.

## Consent

Informed consent was obtained from all participants in this study.

## Conflicts of Interest

The authors declare no conflicts of interest.

## Supporting information


**Table S1:** Differential diagnosis of benign and malignant ovarian tumors by CA 125, HE4, ROMA, CPH‐I, and combination markers.
**Table S2:** Comparative diagnosis efficacy of CA 125, HE4, ROMA, and CPH‐I in early versus advanced‐stage ovarian carcinoma: multimarker algorithm analysis.

## Data Availability

The data that support the findings of this study are available on request from the corresponding author. The data are not publicly available due to privacy or ethical restrictions.
