# Supplementary material for: Application of Serum CA125, HE4, ROMA, and CPH‐I in the Preoperative Differentiation of Ovarian Tumors: A Diagnostic Accuracy Study
Source: J Clin Lab Anal. 2026 Aug 11;40(17):e70319. doi: 10.1002/jcla.70319 (PMC13458866; doi:10.1002/jcla.70319)
Supplement: Supplementary file 1 [file JCLA-40-e70319-s001.docx]

**Supplementary Table S1 Differential diagnosis of benign and malignant ovarian tumors by CA 125, HE4, ROMA, CPH-I, and combination markers**

|  | **SE(%)** | **SP(%)** | **PPV(%)** | **NPV(%)** | **AUC(95%CI)** | **P-value** | **cutoff** |
| --- | --- | --- | --- | --- | --- | --- | --- |
| **Total（n=345）** |  |  |  |  |  |  |  |
| CA125(U/ml) | 67.70 | 94.10 | 90.68 | 77.53 | 0.865(0.825-0.906) | <0.001 | 87.93 |
| HE4(pmol/L) | 81.60 | 90.40 | 87.76 | 85.35 | 0.897(0.860-0.934) | <0.001 | 67.28 |
| ROMA（%） | 84.80 | 91.40 | 89.33 | 87.69 | 0.916(0.883-0.950) | <0.001 | 16.42 |
| CPH-I（%） | 81.60 | 90.90 | 88.36 | 85.43 | 0.924(0.895-0.953) | <0.001 | 4.51 |
| CA125+HE4 | 82.90 | 89.30 | 86.75 | 86.08 | 0.912(0.879-0.945) | <0.001 | NA |
| CA125+HE4+ROMA | 84.80 | 91.40 | 89.33 | 87.69 | 0.919(0.886-0.951) | <0.001 | NA |
| CA125+HE4+CPH-I | 86.70 | 86.10 | 84.05 | 88.46 | 0.925(0.896-0.954) | <0.001 | NA |
| **Premenopausal（n=185）** | |  |  |  |  |  |  |
| CA125(U/ml) | 86.40 | 72.30 | 49.30 | 94.40 | 0.856（0.787-0.925） | <0.001 | 42.17 |
| HE4(pmol/L) | 63.60 | 98.60 | 93.40 | 89.70 | 0.811（0.719-0.903） | <0.001 | 82.085 |
| ROMA（%） | 65.90 | 96.50 | 85.50 | 90.10 | 0.815（0.724-0.906） | <0.001 | 16.26 |
| CPH-I（%） | 77.30 | 89.40 | 69.50 | 92.70 | 0.886（0.824-0.948） | <0.001 | 3.47 |
| CA125+HE4 | 72.70 | 94.30 | 79.90 | 91.70 | 0.858（0.784-0.932） | <0.001 | NA |
| CA125+HE4+ROMA | 72.70 | 92.90 | 76.20 | 91.60 | 0.853（0.774-0.931） | <0.001 | NA |
| CA125+HE4+CPH-I | 75.00 | 88.70 | 67.40 | 91.90 | 0.871（0.799-0.943） | <0.001 | NA |
| **Post-menopause（n=160）** | |  |  |  |  |  |  |
| CA125(U/ml) | 86.00 | 87.00 | 94.30 | 71.50 | 0.908（0.860-0.956） | <0.001 | 33.72 |
| HE4(pmol/L) | 75.40 | 97.80 | 98.80 | 61.60 | 0.901（0.854-0.947） | <0.001 | 111.9 |
| ROMA（%） | 81.60 | 91.30 | 95.90 | 66.70 | 0.928（0.890-0.966） | <0.001 | 35.4 |
| CPH-I（%） | 74.60 | 97.80 | 98.80 | 60.80 | 0.923（0.884-0.963） | <0.001 | 18.28 |
| CA125+HE4 | 71.90 | 100.00 | 100.00 | 58.90 | 0.921（0.881-0.962） | <0.001 | NA |
| CA125+HE4+ROMA | 76.30 | 97.80 | 98.80 | 62.50 | 0.930（0.892-0.968） | <0.001 | NA |
| CA125+HE4+CPH-I | 76.30 | 95.70 | 97.80 | 62.00 | 0.922（0.882-0.962） | <0.001 | NA |

SE: Sensitivity; SP: Specificity; PPV: Positive predictive value; NPV: Negative predictive value; AUC: Area under the curve.

**Supplementary Table S2 Comparative Diagnosis Efficacy of CA 125, HE4, ROMA, and CPH-I in Early versus Advanced-Stage Ovarian Carcinoma: Multimarker Algorithm Analysis**

| **Total（n=158）** | **SE (%)** | **SP (%)** | **PPV (%)** | **NPV (%)** | **AUC (95%CI)** | **P-value** | **cutoff** |
| --- | --- | --- | --- | --- | --- | --- | --- |
| CA125(U/ml) | 80.90 | 64.60 | 83.96 | 59.62 | 0.759(0.677-0.840) | <0.001 | 91.59 |
| HE4(pmol/L) | 86.40 | 56.20 | 81.90 | 64.29 | 0.756(0.672-0.839) | <0.001 | 94.38 |
| ROMA（%） | 82.70 | 66.70 | 85.05 | 62.75 | 0.784(0.703-0.865) | <0.001 | 46.33 |
| CPH-I（%） | 69.10 | 81.20 | 89.41 | 53.42 | 0.787(0.708-0.867) | <0.001 | 45.20 |
| CA125+HE4 | 79.10 | 64.60 | 79.20 | 40.70 | 0.777(0.697-0.857) | <0.001 | - |
| CA125+HE4+ROMA | 82.70 | 66.70 | 85.00 | 62.70 | 0.788(0.708-0.867) | <0.001 | - |
| CA125+HE4+CPH-I | 70.00 | 81.20 | 89.50 | 54.20 | 0.786(0.705-0.866) | <0.001 | - |

SE: Sensitivity; SP: Specificity; PPV: Positive predictive value; NPV: Negative predictive value; AUC: Area under the curve.
